# Cardiac geometry, as assessed by cardiac magnetic resonance, can differentiate subtypes of chronic thromboembolic pulmonary vascular disease

**DOI:** 10.3389/fcvm.2022.1004169

**Published:** 2022-12-13

**Authors:** Michael McGettrick, Helen Dormand, Melanie Brewis, Martin K. Johnson, Ninian N. Lang, Alistair Colin Church

**Affiliations:** ^1^The Scottish Pulmonary Vascular Unit, Golden Jubilee National Hospital, Glasgow, United Kingdom; ^2^Institute of Cardiovascular and Medical Sciences, University of Glasgow, Glasgow, United Kingdom

**Keywords:** thromboembolic disease, chronic thromboembolic pulmonary hypertension, cardiac MRI (CMR), pulmonary embolism, pulmonary hypertension

## Abstract

**Background:**

Ventricular septal flattening reflects RV pressure overload in pulmonary arterial hypertension. Eccentricity index (EI) and pulmonary artery distensibility (PAD) correlate with pulmonary artery pressure. We assessed the utility of these using cardiac magnetic resonance (CMR) to assess for pulmonary hypertension (PH) in patients with chronic thromboembolic disease. This may allow non-invasive differentiation between patients who have chronic thromboembolic pulmonary hypertension (CTEPH) and those with pulmonary vascular obstructions without PH at rest, known as chronic thromboembolic pulmonary disease (CTEPD).

**Methods:**

Twenty patients without resting pulmonary hypertension, including ten with chronic thromboembolic disease, and thirty patients with CTEPH were identified from a database at the Scottish Pulmonary Vascular Unit. CMR and right heart catheter had been performed within 96 h of each other. Short-axis views at the level of papillary muscles were used to assess the EI at end-systole and diastole. Pulmonary artery distensibility was calculated using velocity-encoded images attained perpendicular to the main trunk.

**Results:**

Eccentricity index at end-systole and end-diastole were higher in CTEPH compared to controls (1.3 ± 0.5 vs. 1.0 ± 0.01; *p* ≤ 0.01 and (1.22 ± 0.2 vs. 0.98 ± 0.01; *p* ≤ 0.01, respectively) and compared to those with CTED. PAD was significantly lower in CTEPH compared to controls (0.13 ± 0.1 vs. 0.46 ± 0.23; *p* ≤ 0.01) and compared to CTED. End-systolic EI and end-diastolic EI correlated with pulmonary vascular hemodynamic indices and exercise variables, including mean pulmonary arterial pressure (R0.74 and 0.75, respectively), cardiac output (*R*-value −0.4 and −0.4, respectively) NTproBNP (*R*-value 0.3 and 0.3, respectively) and 6-min walk distance (*R*-value −0.7 and −0.8 respectively). Pulmonary artery distensibility also correlated with 6-min walk distance (*R*-value 0.8).

**Conclusion:**

Eccentricity index and pulmonary artery distensibility can detect the presence of pulmonary hypertension in chronic thromboembolic disease and differentiate between CTEPH and CTED subgroups. These measures support the use of non-invasive tests including CMR for the detection pulmonary hypertension and may reduce the requirement for right heart catheterization.

## Introduction

Chronic thromboembolic pulmonary hypertension (CTEPH), classed as Group IV in the 2018 National Institute for Health and Care Excellence classification of pulmonary hypertension (1), is a progressive condition and associated with a high symptom burden (2). It is currently defined by a mean pulmonary arterial pressure ≥25 mmHg and a pulmonary capillary wedge pressure <15 mmHg, as measured by right heart catheterization, in addition to at least one persistent perfusion defect seen on imaging (V/Q scanning or pulmonary angiography) after 3 months of anticoagulation. It usually occurs following acute pulmonary embolus (3).

The clinical consequences of CTEPH include impaired exercise capacity, right heart failure and premature death (4). The management of CTEPH is well validated (5). Indeed, surgical pulmonary endarterectomy has been shown to cure, or substantially reduce the pulmonary arterial and right ventricular pressures in those with central disease (5). In comparison, chronic thromboembolic disease (CTED) [now also known chronic thromboembolic pulmonary disease (CTEPD)] (6), is characterized by chronic arterial obstruction, without evidence of pulmonary hypertension at rest. These patients may have functional limitation identified by cardiopulmonary exercise testing (CPET) (4, 7, 8) and, in certain cases, treatment leading to symptomatic improvement with PEA has been performed (9). However, there is no accepted medical or surgical therapy for this group.

Therefore, diagnosis of pulmonary hypertension is key in differentiating between CTEPH and CTED and therefore making a difference to the treatment offered. Echocardiography remains the main screening investigation for pulmonary hypertension. However, there are limitations in obtaining good image windows can often render this technique difficult in many patients, for example those who are obese. Right heart catheterization (RHC) remains the gold standard diagnostic technique for pulmonary hypertension (10) but this is invasive and carries some associated risks and discomfort for patients. A non-invasive technique which is able to discern between thrombotic groups is needed.

Cardiac MRI is employed with increasing frequency for the assessment of patients with pulmonary arterial hypertension because of increased availability, ease of use and shortened acquisition times (11). CMR has several advantages over echocardiography, including better reproducibility in the assessment of ventricular mass, volume and ejection fraction, and is the gold standard for non-invasive assessment of the right ventricle (RV) (12). Whilst CMR is used to measure transvalvular pressure gradients, its use in measuring RV and pulmonary artery pressures has been more limited. EI is defined as the ratio of the length of two perpendicular minor-axis at end-systolic and end-diastolic Left Ventricular diameters, one of which is bisected and lies perpendicular to the interventricular septum (13). This measurement has recently been validated using echocardiography for use in pediatric patients with pulmonary hypertension and in adults with IPAH (14). To our knowledge, this has not been formally assessed in patients with CTED or CTEPH using CMR.

We hypothesized that EI would be abnormal in CTEPH and normal in CTED and that we could use this investigation to differentiate between normal and disease states that would benefit from medical/surgical treatment. Evaluation of pulmonary artery distensibility and peak pulmonary blood flow in these groups were also undertaken. We correlated these findings with right heart catheterization hemodynamic indices, functional capacity using 6-min walk distance, cardiopulmonary exercise testing and cardiac biomarker, N-terminal pro B-type natriuretic peptide (NT-proBNP).

## Materials and methods

This study was approved by the East of England—Cambridge Central Research Ethics committee (20/EE/0195).

**FIGURE 1:**
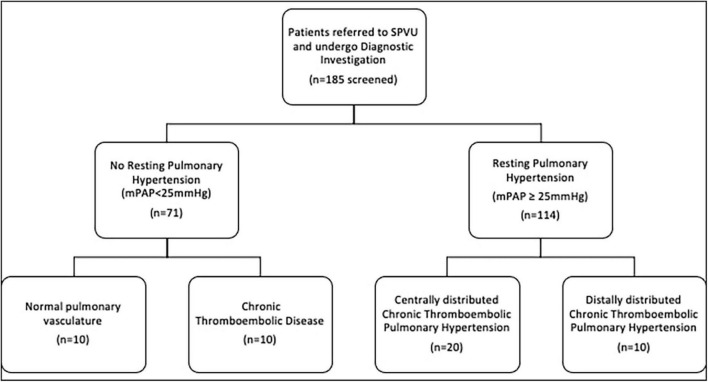
Consort diagram of patient inclusion into the analysis.

Patients referred for investigation of potential CTEPH and CTED between January 2016 and December 2019 were identified from a clinical database at the Scottish Pulmonary Vascular Unit (SPVU), Glasgow, UK. Recruitment of patients is summarized in Figure 1. Diagnostic investigations for patients at the SPVU include contemporaneous CMR, CPET and right heart catheterization. Patients were excluded if they had significant obstructive lung disease (FEV1: FVC < 70%), untreated sleep apnea, atrial fibrillation, a history of ischemic heart disease, left ventricular ejection fraction of <50% by CMR analysis, structural heart disease (valvular heart disease and those with congenital systemic to pulmonary shunts) and those patients already taking pulmonary vasodilators at the time of diagnosis.

Consecutive patients with complete data sets were included in the analysis (Figure 1). Over that period, 71 patients underwent detailed investigation and had no evidence of pulmonary hypertension at rest. Of these, 23 were diagnosed with CTED, 10 of whom had complete data sets of radiological and physiological investigations, including CPET. The main limiting factor was access to CPET data as this relies on a good degree of exercise capacity to generate meaningful results. Those with distal CTEPH were functionally limited, so CPET data was often not available for these patients. The remaining 48 patients had normal pulmonary vasculature, 10 of whom had complete data sets of radiological and physiological investigations. One hundred fourteen patients underwent investigations for, and were diagnosed with, CTEPH, thirty of whom were included in analysis due to data availability. Twenty of these patients were seen to have central disease, defined as the presence of thrombus in the segmental pulmonary arteries. Ten had distal disease, which was non-operable.

### Cardiac magnetic resonance imaging

**FIGURE 2:**
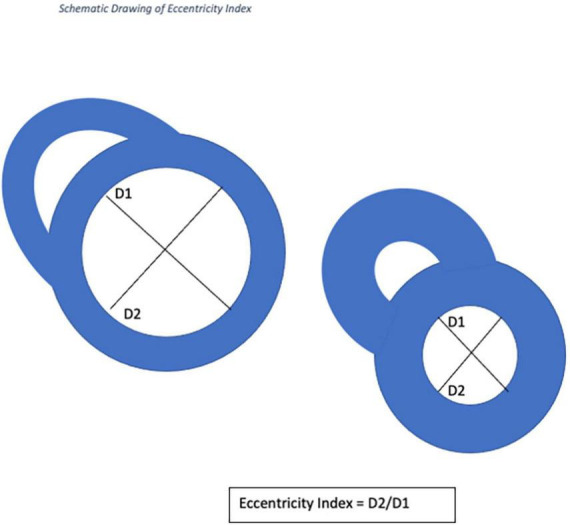
Schematic drawing of eccentricity index calculation during systole and diastole.

**FIGURE 3:**
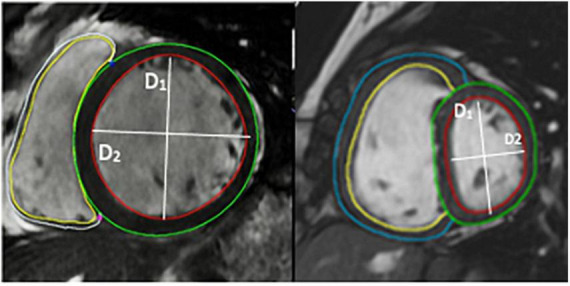
Image of short axis CMR demonstrating the volumetric analysis and the calculation of eccentricity index using CMR images.

Cardiac magnetic resonance imaging was performed using a 1.5-T magnetic resonance imaging scanner (Sonata Magnetom, Siemens, Erlangen, Germany) in the headfirst supine position, with gated cardiac synchronization. Initial scout images were acquired using fast imaging with steady state precession. Vertical and horizontal long axis cines were planned and acquired based on the scout images. The short axis images were then planned from the horizontal long axis views. RV and LV volumes were determined by automatic tracing of endocardial borders of the short axis stack using Circle Cardiovascular imaging software (Calgary, Canada). All borders were manually verified and corrected as needed. Main pulmonary artery flow curve was captured using velocity-encoded (VENC) CMR, perpendicular to the pulmonary artery. A VENC of 100 cm/s was used for image acquisition. EI was measured at end systole and end diastole in both left and right ventricles (RV) at mid-ventricular level—defined as the level of the papillary muscles. The EI is defined as the ratio of the length of the two perpendicular minor-axis diameters, one of which is bisected and was perpendicular to the interventricular septum (Figures 2, 3). The ratio of the minor axis dimensions was measured, D2/D1, where D1 is ventricular diameter perpendicular to the septum and D2 is ventricular diameter parallel to the septum. In the absence of RV pressure overload, the LV is circular, and D2/D1 would be expected to be 1.0 (13). Main pulmonary artery distensibility was determined using VENC images with luminal area measurements at the moment of maximal flow and the moment of isovolumetric contraction to calculate the ratio of (PA maximum area–PA minimum area)/PA minimum area. Flow curves were generated using the VE-CMR images obtained perpendicular to the pulmonary artery during breath hold.

D1 is the endocardial measurement perpendicular to the interventricular septum at the largest diameter. D2 is the endocardial measurement at the largest length. D2/D1 is used to calculate eccentricity index in end-diastole and end-systole. These can be difficult to define in pulmonary hypertension, so, using the QRD complex to identify systole, images were identified around the end of systole and the end diastole. These were analyzed and those with the smallest LV volume were classed as end-systole and those with the largest were classed as end-diastole.

### Cardiopulmonary exercise testing

Cardiopulmonary exercise testing was performed using an incremental cycle ergometer. The ramp rate was chosen by the physiologist, based on participants’ self-reported fitness, with the aim of exercising for 8–12 min. We used the indices of low peak VO_2_ and high VE/V_CO2_ to assess the level of functional limitation.

### Invasive hemodynamic measurement

Right heart catheterization was performed. Hemodynamic parameters including right atrial, right ventricular, pulmonary artery and pulmonary artery wedge pressure were all measured at rest. Cardiac output was measured using the thermodilution method and pulmonary vascular resistance (PVR) calculated. CMR, CPET and right heart catheterization were performed with a maximum of 3 days between procedures.

Diagnosis was agreed by consensus at the multidisciplinary team meeting at the Scottish Pulmonary Vascular Unit (SPVU) and by the national pulmonary thromboendarterectomy team at Royal Papworth Hospital, Cambridge, UK.

### Statistical analysis

Unpaired *t*-test was used to compare normal distributed variables. Mann Whitney *U* test was used to compare non-normally distributed variables. Spearman correlation was used to assess for correlations between investigations. Analysis of Variance (ANOVA) was used to assess for differences between more than two groups. Receiver Operator Curves were used to identify thresholds of clinical sensitivity and specificity for the investigations. Twenty percentage of images were reviewed by a second observer (>10 years experience), who was blinded to the diagnosis and RHC values, and Pearson’s correlation is used to assess for agreement between continuous variables. A *p*-value < 0.05 was taken to represent statistical significance. Data were analyzed using Microsoft Excel and GraphPad Prism (San Diego, USA, Version 9).

## Results

We identified 30 patients recently diagnosed with CTEPH and 20 control patients with normal resting pulmonary artery values who had a complete data set of investigations. 10 of those with CTEPH were deemed to have distal CTEPH and 20 with proximal disease. There were no significant differences in age and sex between these groups.

### Baseline characteristics

Baseline characteristics are shown in Table 1. Ten of the twenty patients with no pulmonary hypertension at rest had evidence of chronic thromboembolic disease on imaging while the other ten had no evidence of residual thromboembolic disease. As expected, mean (SD) pulmonary arterial pressure (40.2 ± 11.4 vs. 17 ± 4.4 mmHg; *p* ≤ 0.01), PVR (8.5 ± 4.7 vs. 1.9 ± 1.2 Wu; *p* ≤ 0.01) were higher in patients with CTEPH vs. controls. Pulmonary artery wedge pressure was normal in both groups. NTpro-BNP was significantly higher in patients with CTEPH vs. non-PH (1,004 ± 1,291 vs. 184 ± 155 pg/ml; *p* ≤ 0.01).

**TABLE 1:** Baseline variables between those with and without pulmonary hypertension.

Index	Non-PH, *n* = 20 Mean (SD)	CTEPH, *n* = 30 Mean (SD)	*P*-value^+^
Age (years)	53.9 (17.5)	52.2 (15.9)	0.73
Sex (M)	8	16	0.36
BMI (kg/m^2^)	32.3 (6.0)	28.4 (5.6)	0.03
RAP (mmHg)	3.2 (2.3)	6.0 (3.9)	<0.01
RVEDP (mmHg)	5.3 (2.6)	8.8 (4.3)	<0.01
mPAP (mmHg)	17 (4.4)	40.2 (11.4)	<0.01
PAWP (mmHg)	8.3 (3.4)	7.5 (3.6)	0.46
CO (mmHg)	5.1 (1.1)	4.1 (0.9)	<0.01
CI (L/min/m^2^)	2.5 (0.6)	2.1 (0.4)	<0.01
PVR (Wood units)	1.9 (1.2)	8.5 (4.7)	<0.01
S_v_O_2_ (%)	73.1 (4.2)	63.5 (6.8)	<0.01
NTpro-BNP (pg/ml)	184 (155)	1,004 (1,291)	0.01
6MWT (m)	407 (115)	389 (95)	0.55

^+^Unpaired *t*-test. BMI, body mass index; RAP, right atrial pressure; EDP, end diastolic pressure; mPAP, mean pulmonary artery pressure; PAWP, pulmonary artery wedge pressure; CO, cardiac output; CI, cardiac index; BSA, body surface area; PVR, pulmonary vascular resistance; S_v_O_2_, mixed venous saturations.

Cardiopulmonary exercise testing parameters are outlined in Table 2, using the main indices that are used to detect and measure exercise capacity in pulmonary hypertension. There was no significant difference in peak VO_2_ between groups but V_E_/V_CO2_ (49.6 vs. 34.3; *p* ≤ 0.01) was higher in CTEPH, in keeping with increased dead-space ventilation.

**TABLE 2:** Baseline cardiopulmonary exercise tests between those with and without resting pulmonary hypertension.

	Non-PH, *n* = 20 Mean (SD)	CTEPH, *n* = 20 Mean (SD)	*P*-value^+^
Peak VO_2_ ml/kg/min	1.3 (0.44)	5.28 (19.4)	0.15
V_E_/V_CO2_	34.3 (6.9)	49.6 (11.5)	<0.01

^+^Unpaired *t*-test performed for normal distributed variables and Mann Whitney *U* performed for others.

### Differences in cardiac magnetic resonance analysis between chronic thromboembolic pulmonary hypertension and non-chronic thromboembolic pulmonary hypertension groups

Differences in the novel geometric CMR indices in those with pulmonary hypertension and those without are shown in Table 3. Pulmonary artery distensibility was significantly lower in those with CTEPH (0.13 ± 0.1 vs. 0.46 ± 0.23; *p* ≤ 0.01) with a significantly higher EI in systole (1.3 ± 0.5 vs. 1.0 ± 0.01; *p* ≤ 0.01) and diastole (1.22 ± 0.2 vs. 0.98 ± 0.01; *p* ≤ 0.01) compared to the control group. We showed that PA diastolic area was significantly higher in the pulmonary hypertension group (5.26 ± 1.42 vs. 8.13 ± 1.9; *p* ≤ 0.01). There was no significant difference in peak pulmonary artery blood flow velocity.

**TABLE 3:** Novel measurements by cardiac MRI in patients with chronic thromboembolic pulmonary hypertension vs. those without.

Index	Non-PH, *n* = 20 Mean (SD)	CTEPH, *n* = 30 Mean (SD)	*P*-value^+^
Distensibility	0.46 (0.23)	0.13 (0.1)	<0.001
Systolic LVEI	1.0 (0.01)	1.3 (0.5)	<0.001
Diastolic LVEI	0.98 (0.01)	1.22 (0.2)	<0.001
Peak blood flow (mls/s)	386.1 (60)	365.1 (104)	0.23
PA diastolic area (cm^2^)	5.26 (1.42)	8.13 (1.9)	<0.001

^+^Mann Whitney *U* test. cm^2^, centimeters squared; LVEI, left ventricular eccentricity index; mls/s, milliliters per second.

Mean pulmonary artery pressure correlated with systolic LVEI (*R*-value 0.74, *p* ≤ 0.01) and with diastolic LVEI (*R*-value 0.75, *p* ≤ 0.01). Systolic and diastolic EI also correlated with prognostically important markers including PVR (*R*-value 0.75, and 0.76, respectively), mixed venous saturations (*R*-value −0.65, −0.68, respectively) and 6-min walk distance (*R*-values −0.71 and −0.78, respectively) (Table 4 and Supplementary Figure 1).

**TABLE 4:** Spearman correlation of standard pulmonary hypertension indices vs. novel cardiac MRI indices.

Index	Systolic LVEI *R*-value (*P*-value)	Diastolic LVEI *R*-value (*P*-value)	Distensibility *R*-value (*P*-value)	Peak blood flow *R*-value (*P*-value)
RAP (mmHg)	0.32 (0.02)	0.32 (0.02)	−0.11 (0.43)	−0.07 (0.61)
EDP (mmHg)	0.31 (0.02)	0.36 (<0.01)	−0.08 (0.59)	−0.12 (0.41)
sPAP (mmHg)	0.72 (<0.01)	0.73 (<0.01)	−0.3 (0.03)	−0.33 (0.02)
dPAP (mmHg)	0.74 (<0.01)	0.77 (<0.01)	−0.20 (0.16)	−0.34 (0.02)
mPAP (mmHg)	0.74 (<0.01)	0.75 (<0.01)	−0.27 (0.06)	0.33 (0.02)
PAWP (mmHg)	−0.06 (0.69)	−0.03 (0.86)	0.05 (0.72)	0.05 (0.71)
CO (mmHg)	−0.44 (<0.01)	−0.40 (<0.01)	−0.03 (0.8)	0.46 (<0.01)
CI (L/min/m^2^)	−0.43 (<0.01)	−0.41 (<0.01)	−0.09 (0.53)	0.33 (0.02)
PVR (Wu)	0.72 (<0.01)	0.72 (<0.01)	−0.21 (0.14)	−0.40 (<0.01)
S_v_O_2_ (%)	−0.65 (<0.01)	−0.68 (<0.01)	0.15 (0.3)	0.61 (<0.01)
NTpro-BNP (pg/ml)	0.34 (0.03)	0.28 (0.08)	−0.38 (0.02)	−0.04 (0.83)
6MWT (m)	−0.71 (0.01)	−0.78 (<0.01)	0.77 (<0.01)	0.18 (0.29)

RAP, right atrial pressure; EDP, end diastolic pressure; sPAP, systolic pulmonary artery pressure; dPAP, diastolic pulmonary artery pressure; mPAP, mean pulmonary artery pressure; PAWP, pulmonary artery wedge pressure; CO, cardiac output; CI, cardiac index; BSA, body surface area; PVR, pulmonary vascular resistance; S_v_O_2_, mixed venous saturations; NTpro-BNP, N-terminal pro natriuretic peptide; 6MDT, 6-min walk test distance.

Those with distal CTEPH in our group were unable to perform cardiopulmonary exercise testing due to physical limitations, and, as such there is no data available for this group. CPET data for the other three groups are outlined in Table 5. Whilst there is a reduction in peak VO_2_ in the CTEPH group, this did not reach statistical significance. However, there was a progressive rise in the V_E_/V_CO2_ (*p* ≤ 0.01), consistent with greater dead-space ventilation in those with CTEPH.

**TABLE 5:** Baseline cardiopulmonary exercise testing between subgroups of thrombotic pulmonary vascular disease.

	Normal (*n* = 10)	CTED (*n* = 10)	Proximal CTEPH (*n* = 20)	*p*-value^+^
VO_2_ ml/kg/min	1.3 (0.5)	1.3 (0.4)	1 (0.5)	0.21
V_E_/V_CO2_	31.7 (5.2)	36.9 (7.7)	49.67 (11.81)	<0.001

^+^Unpaired *t*-test performed for normal distributed variables and Mann Whitney *U* performed for others.

We have shown good interobserver agreement in our CMR determinants, lending confidence to our results using Pearson’s correlation (*R* = 0.9).

### Determining threshold values for detection of pulmonary hypertension

**FIGURE 4:**
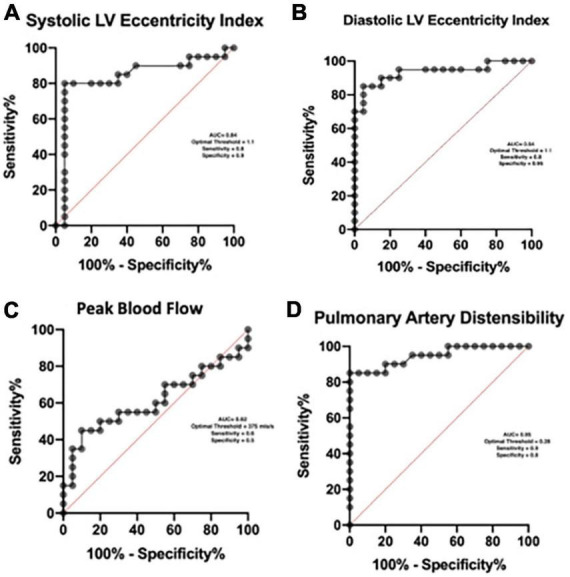
ROC Curves used to identify diagnostic thresholds for novel CMR measurements.

Using Receiver operator curves the optimal threshold for the detection of pulmonary hypertension with the systolic EI is 1.1 (AUC = 0.84), diastolic index is 1.1 (AUC = 0.94) and pulmonary artery distensibility is 0.28 (AUC = 0.95). We found no meaningful threshold for the peak blood flow (Figure 4).

### Cardiac magnetic resonance can differentiate between subgroups of thrombotic pulmonary vascular disease

Patients without pulmonary hypertension were divided into two groups—those without evidence of pulmonary vascular obstruction on CT and V/Q imaging (normal controls), and those with residual radiological proven disease. Differences in the hemodynamic indices across those who had normal pulmonary vasculature, those with CTED and those with proximal and distal CTEPH were compared (Table 6). As expected across the spectrum of thrombotic pulmonary vascular disease, there was a progressive rise in the mean pulmonary artery pressure (*p* ≤ 0.01), a progressive reduction in the cardiac index (*p* ≤ 0.01) and rise in the PVR (*p* ≤ 0.01).

**TABLE 6:** ANOVA of the hemodynamic values of across all patient groups.

Index	Normal Mean (SD) *n* = 10	CTED Mean (SD) *n* = 10	Proximal CTEPH Mean (SD) *n* = 20	Distal CTEPH Mean (SD) *n* = 10	*P*-value^+^
Age (years)	46 (18.9)	61.8 (12.2)	51.9 (16.2)	65.7 (9.3)	0.01
BMI (kg/m^2^)	30.4 (5)	34.1 (6.5)	28.5 (5.7)	29.7 (4.3)	0.08
RAP (mmHg)	3.4 (2.8)	2.9 (1.9)	6.3 (3.9)	5.6 (4.1)	0.04
EDP (mmHg)	6.2 (2.8)	4.3 (2.2)	8.9 (4.4)	8.7 (4.4)	0.01
mPAP (mmHg)	14.1 (3.4)	19.9 (3.1)	41.1 (13.5)	38.3 (4.4)	<0.001
PAWP (mmHg)	7.8 (3.0)	8.7 (3.9)	7.4 (3.4)	7.7 (4.1)	0.82
CO (mmHg)	5.6 (1.1)	4.5 (0.8)	4.3 (0.9)	3.9 (0.8)	<0.001
CI (CO/BSA)	2.9 (0.6)	2.2 (0.2)	2.2 (0.4)	2.0 (0.4)	<0.001
PVR (Wu)	1.2 (0.9)	2.5 (1.1)	8.6 (4.5)	8.3 (2.7)	<0.001
S_v_O_2_ (%)	74.7 (5.1)	71.5 (2.2)	64.45 (6.7)	61.6 (7.0)	<0.001

BMI, body mass index; RAP, right atrial pressure; EDP, end diastolic pressure; mPAP, mean pulmonary artery pressure; PAWP, pulmonary artery wedge pressure; CO, cardiac output; CI, cardiac index; PVR, pulmonary vascular resistance; S_v_O_2_, mixed venous saturations.

^+^Unpaired *t*-test performed for normal distributed variables and Mann Whitney *U* performed for others.

**FIGURE 5:**
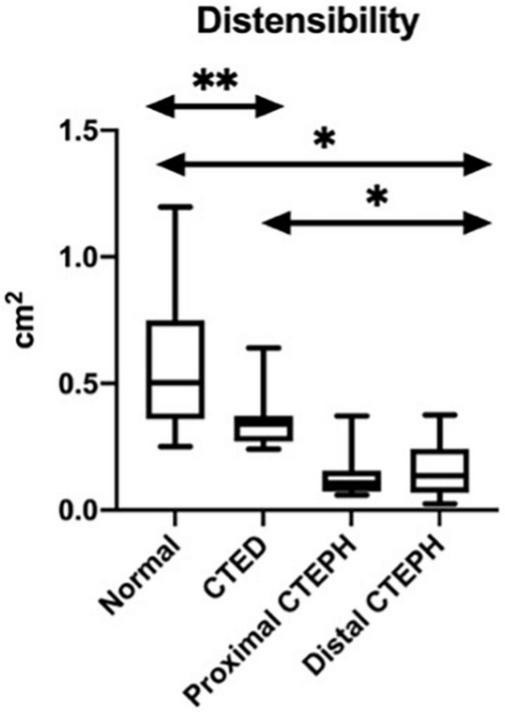
Box and whisker plots for the variations in the novel CMR indices between thromboembolic disease subtypes. *p < 0.01, ^**^p < 0.05.

The differences between groups in the geometric novel CMR indices are shown in Figure 5. There was a significant difference between those with both distal and proximal CTEPH and CTED in the systolic LEVI (*p* ≤ 0.01), diastolic LVEI (*p* ≤ 0.01), and pulmonary artery distensibility (*p* ≤ 0.01). No significant differences between subgroups in peak blood flow were seen.

### Subgroup analysis in patients with thrombotic pulmonary vascular disease with no pulmonary hypertension

The differences in baseline variables and novel CMR indices between those without evidence of pulmonary vascular obstruction on CT and V/Q imaging (normal controls), and those with residual radiological proven disease were evaluated (Table 7). Age and sex were similar between groups. Although the mean pulmonary artery pressures and PVR were within the normal range for both groups, mPAP [19.9 (3.1) vs. 14.1 (3.4) mmHg; *p* ≤ 0.01] and PVR [2.5 (1.1) vs. 1.2 (0.9) WU; *p* = 0.01] were higher in the CTED group, in keeping with pulmonary vascular occlusions. In addition, there was a significantly lower pulmonary artery distensibility [0.34 (0.1) vs 0.5 (0.4); *p* = 0.03] and a significantly higher LVEI in systole [1.04 (0.03) vs. 0.99 (0.08); *p* ≤ 0.01] and diastole [1 (0.1) vs. (0.96 (0.07); *p* = 0.02] in the CTED group compared to normal but no significant difference in peak blood flow between these groups. Threshold analysis for identifying the presence of CTED using systolic and diastolic LVEI is 1.0 and pulmonary artery distensibility is 0.37 (Supplementary Figure 2).

**TABLE 7:** Baseline characteristics of normal controls vs. those with chronic thromboembolic disease.

Index	Normal (*n* = 10) Mean (SD)	CTED (*n* = 10) Mean (SD)	*P*-value^+^
Age (years)	46 (18.9)	62 (12.2)	0.04
Sex (Male)	4	4	0.65
BMI (kg/m^2^)	30.4 (5)	34.1 (6.5)	0.17
RAP (mmHg)	3.4 (2.8)	2.9 (1.9)	0.64
mPAP (mmHg)	14.1 (3.4)	19.9 (3.1)	<0.01
PAWP (mmHg)	7.8 (3)	8.7 (3.9)	0.57
CO (mmHg)	5.6 (1.1)	4.5 (0.8)	0.02
CI (L/min/m^2^)	2.9 (0.6)	2.2 (0.25)	<0.01
PVR (Wu)	1.2 (0.9)	2.5 (1.1)	0.01
S_v_O_2_ (%)	74.7 (5.1)	71.5 (2.2)	0.09
Distensibility	0.57 (0.3)	0.35 (0.1)	0.03
Systolic LVEI	0.97 (0.04)	1.03 (0.04)	<0.01
Diastolic LVEI	0.96 (0.03)	1.02 (0.05)	0.02
Peak blood flow (mls/s)	384.3 (64)	387.8 (59.3)	0.97
PA diastolic area (cm^2^)	5.07 (1.6)	5.45 (1.28)	0.78

^+^Unpaired *t*-test performed for normal distributed variables, Mann Whitney *U* performed for others. BMI, body mass index; RAP, right atrial pressure; EDP, end diastolic pressure; mPAP, mean pulmonary artery pressure; PAWP, pulmonary artery wedge pressure; CO, cardiac output; CI, cardiac index; PVR, pulmonary vascular resistance; S_v_O_2_, mixed venous saturations; cm^2^, centimeter squared; LVEI, left ventricular eccentricity index; mls/s, milliliters per second.

## Discussion

In this retrospective cohort study of patients, we have demonstrated three important findings.

• That elevated EI, both in systole and diastole, and reduced pulmonary artery distensibility are predictive of the presence of pulmonary hypertension in CTEPH.
• Both EI and pulmonary artery distensibility could discriminate between the normal, CTED and CTEPH population.
• Both systolic and diastolic EI correlate with clinical markers of outcome.

Furthermore, using a CMR EI threshold of 1.1 has a high sensitivity and specificity for the identification of the presence of pulmonary hypertension. To our knowledge, this is the first time the utility of this measurement has been demonstrated in thrombotic pulmonary vascular disease with CMR. In addition, we have demonstrated that lower pulmonary artery distensibility is predictive of the presence of pulmonary hypertension in patients with radiological features of thromboembolic disease. However pulmonary arterial peak blood flow was not useful for the detection of PH. We acknowledge that there has been a change to the hemodynamic values in the definition of pulmonary hypertension in the 6th World Symposium definition. We have chosen to use the 5th World symposium definition because existing evidence for therapy Is based on these criteria, lending more clinical relevance to our findings in detecting patients who may be amenable to treatment options.

Cardiac magnetic resonance is the gold standard investigation for assessment of right ventricular function and has the added benefit over echocardiogram of allowing detection of pulmonary vascular occlusion with magnetic resonance pulmonary angiogram. Of additional clinical significance and importance is trying to differentiate patients who have CTED or CTEPH using non-invasive modalities. This is important because management can be different and currently many patients with CTED require right heart catheterization in order to differentiate. The data shown in this paper suggests that patients with vascular obstructions on CT can be differentiated on the basis of presence of PH by using certain CMR thresholds of EI (1.1) and pulmonary artery distensibility (0.28). This is important as currently patients with CTED do not have any therapeutic options and therefore do not routinely need to undergo any invasive investigations. If the patient can manage, using CPET is a useful adjunct in the non-invasive assessment of these patients.

Both systolic and diastolic EI correlate with pulmonary artery pressure measurements. This finding is likely explained by the increased pulmonary artery pressure leading to septal flattening and impaired filling of the left ventricle. A larger index reflects more severe disease, as was shown using echocardiography in a pediatric population by Burkett et al. (14). Both S_v_O_2_ (15) and NTpro-BNP (16) have been linked to prognosis in CTEPH and there is a direct correlation between these surrogate markers of RV function and EI and pulmonary artery distensibility. Further work is required to demonstrate that the novel MRI indices may also be useful as a prognostic outcome measure in CTEPH.

Our findings confirm other studies demonstrating that an abnormal EI can be used to accurately define clinically important pulmonary hypertension (13, 14). Ryan et al. (13) and Burkett et al. (14) have demonstrated the use of this measurement in IPAH. However these studies used echocardiography to determine this. In addition, our findings are in agreement with Malhotra et al. that pulmonary artery distensibility can be used to identify clinically relevant pulmonary hypertension (17), although this was mainly in patients with left heart disease. Our study is also in keeping with Lau et al. who have shown that loss of distensibility Is an early sign of pulmonary vascular disease (18). However, this study adds to the literature by demonstrating that these findings also relate to CTEPH and importantly can differentiate between CTED and CTEPH.

In pulmonary hypertension, there is often dyssynchrony between right and left ventricular onset of diastole. At early LV diastole, the pressure in the RV remains significantly elevated, causing septal displacement to the left side. Systole represents the smallest LV size in the cardiac cycle and, as such, this point captures the maximal septal wall displacement. These results show that the resulting differences in EI can be a useful measurement to screen for pulmonary hypertension. In the normal pulmonary artery, systole causes the wall to stretch in order to accommodate the cardiac output, without much rise in the pulmonary artery pressure. In the early stages of pulmonary hypertension, the pressure will rise slowly as there is some capacity for the pulmonary artery to compensate by dilating. However, in more advanced disease, once the vessel has reached its maximal stretch capacity, the pressure will increase more rapidly. There is then little change in the vessel diameter between systole and diastole, which is demonstrated by reduced distensibility. We have demonstrated that those with CTED also have a reduced distensibility, reflective of the abnormal underlying pulmonary vasculature. This may be a more sensitive and clinically relevant measurement in order to detect CTED and has previously been demonstrated in patients with group II pulmonary hypertension (17). In group II disease, a lower distensibility was an early sign of pulmonary hypertension. When the blood vessel is at its maximal diameter, such as in later stage pulmonary hypertension, it is difficult to detect subtle progression in the pulmonary vascular disease, and, as such, EI may be a more sensitive predictor of disease progression.

It is clinically important to differentiate those patients with CTED and normal patients. While there was a higher mean pulmonary artery pressure value in the CTED population relative to normal patients, these values lay at the upper end of the normal range. This may make it difficult to differentiate from normal. However, this study demonstrated that a significantly reduced pulmonary artery distensibility in the CTED population could differentiate between normal and CTED. Given that the LVEI is proportionate to pressure in the pulmonary vasculature, the changes in the LVEI in the CTED patients compared to the normal population is likely explained by the fact the patients with CTED had a mean mPAP of 19.9 mmHg, which is the upper limit of normal (1). Those with normal pulmonary vasculature had a mean mPAP of 14.1 mmHg, which explains their normal LVEI. Indeed recent studies have suggested lowering the diagnostic value for PH to a mPAP of greater than 20 mmHg as it is now recognized that the normal mPAP is around 14 mmHg (3).

This study has a number of strengths. There were no significant differences between age and sex in our patient groups, with the baseline hemodynamic variables that would be expected in the relative populations of normal, CTED and CTEPH. We have complete datasets on all of the patients included in the study and all of the investigations contemporaneously (median 3 days). This contrasts with other studies where imaging has been performed at a different time point from the right heart catheterization (19, 20). We recognize some limitations with this study including its retrospective nature. In addition, although we have attempted to use data from as large a cross section of population as is possible from our single center, the numbers remain limited and, as such, further studies are merited to validate our findings. We have included consecutive patients with central CTEPH who were able to undergo CPET, and we recognize this excludes a number of patients. We recognize that further work is required to fully determine if CMR is robust enough to differentiate the spectrum of chronic thromboembolic disease with or without pulmonary hypertension without the need for invasive investigation. We have focused on well validated CPET indices used in pulmonary hypertension, VO_2_ max and VE/V_CO2_, but have not assessed the use of anaerobic threshold, which has been quantifying the severity of left heart disease. Future studies could include this as part of analysis to evaluate the use in this population.

However, this paper confirms that CMR parameters can be used in conjunction with other invasive and non-invasive investigations to evaluate patients who are suspected as having pulmonary vascular disease and allow clinicians to establish the diagnosis and determine appropriate management. This is an important addition to the literature on the management of these conditions.

## Conclusion

In this retrospective cohort study of patients, we have demonstrated three important findings. Firstly, that elevated EI, both in systole and diastole, and reduced pulmonary artery distensibility are predictive of the presence of pulmonary hypertension in CTEPH. Secondly, both EI and pulmonary artery distensibility could discriminate between the normal, CTED and CTEPH population, and finally, both systolic and diastolic EI correlate with clinical markers of outcome. These have not been demonstrated before in chronic thromboembolic pulmonary vascular disease using CMR.

## Data availability statement

The raw data supporting the conclusions of this article will be made available by the authors, without undue reservation.

## Ethics statement

This study was approved by the East of England—Cambridge Central Research Ethics committee (20/EE/0195). Written informed consent for participation was not required for this study in accordance with the national legislation and the institutional requirements.

## Author contributions

MM was the main author of the manuscript. HD contributed to the gathering of non-invasive measurements. MB and MJ contributed to the gathering of invasive and non-invasive measurements. NL reviewed the manuscript before submission. AC contributed to the gathering of invasive and non-invasive measurements and review of the manuscript. All authors have read and approved the manuscript.
