# Supplementary material for: Cardiac geometry, as assessed by cardiac magnetic resonance, can differentiate subtypes of chronic thromboembolic pulmonary vascular disease
Source: Front Cardiovasc Med. 2022 Dec 13;9:1004169. doi: 10.3389/fcvm.2022.1004169 (PMC9793745; doi:10.3389/fcvm.2022.1004169)
Supplement: Supplementary file 1 [file Table_1.docx]

***Using Cardiac Magnetic Resonance to Assess Cardiac Geometry in the diagnosis of Chronic Thromboembolic Pulmonary Vascular Disease***

*M McGettrick, H Dormand, M Brewis, N N Lang, M Johnson, C Church*

**Supplementary Data**

All Affiliated with

*The Scottish Pulmonary Vascular Unit*

*Golden Jubilee National Hospital*

*Glasgow, UK*

*G81 4DY*

Figure 1 supp. Correlation of standard Pulmonary hypertension haemodynamics versus novel cardiac MRI indices
mPAP – mean Pulmonary Artery Pressure; PVR – Pulmonary Vascular Resistance; SvO2 – Mixed venous saturations; 6MDT – 6-minute walk test distance

| Index | Systolic LVEI | Diastolic LVEI* |
| --- | --- | --- |
| mPAP (mmHg) | 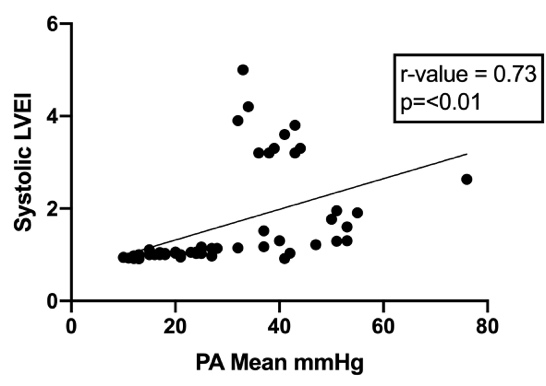 | 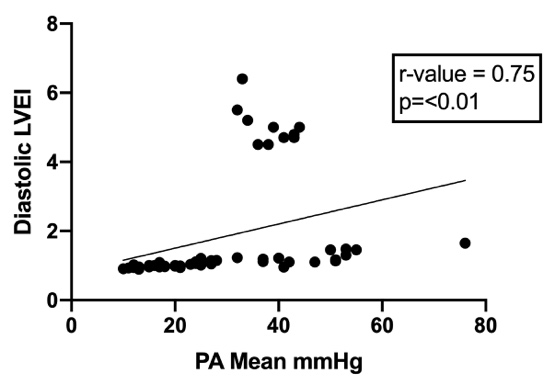 |
| PVR (Wu) | 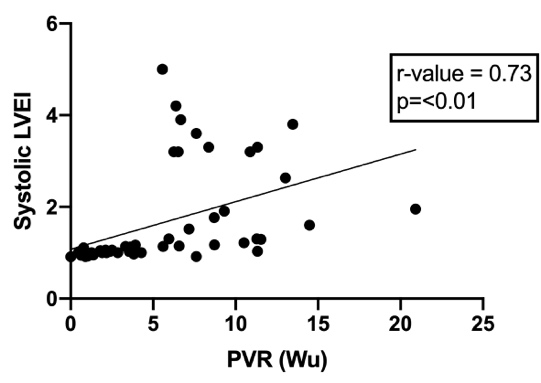 | 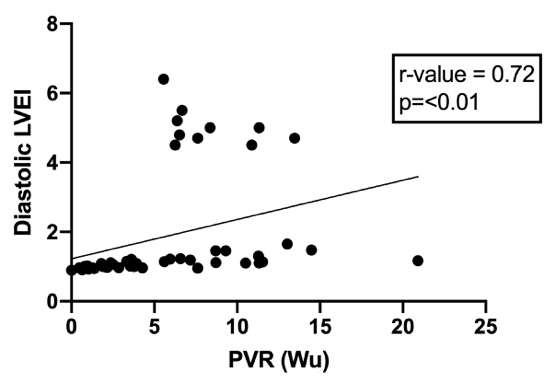 |
| SvO_2_(%) | 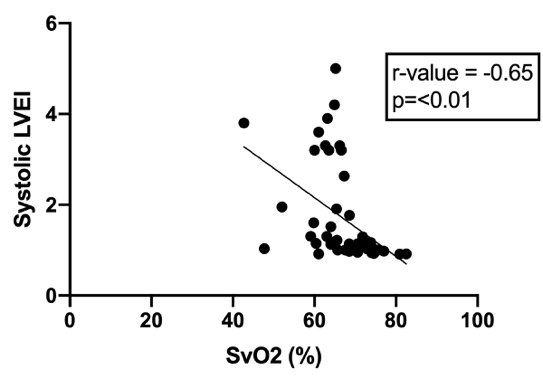 | 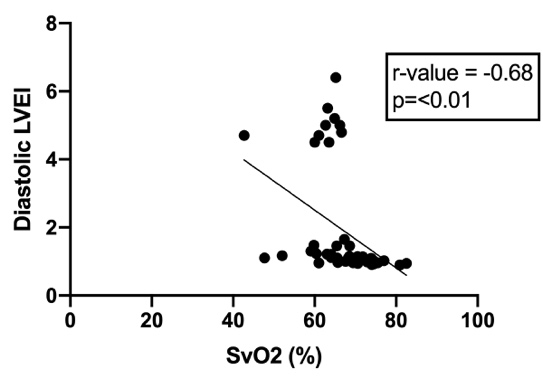 |
| 6MWT (m) | 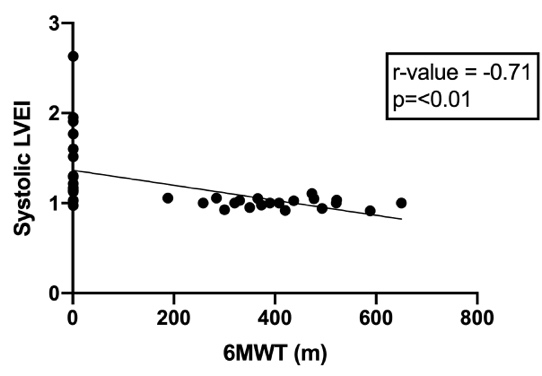 | 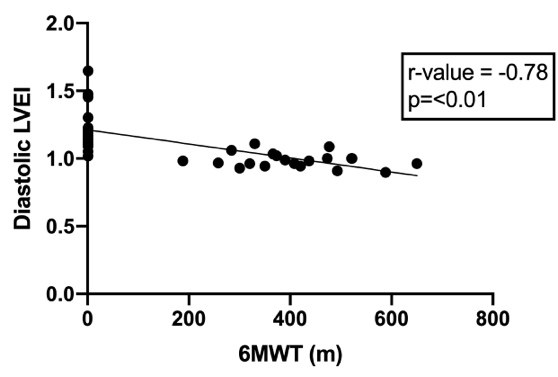 |

Figure 2 supp. Normal vs CTED Roc curves for novel cardiac MRI indices
CTED – chronic thromboembolic disease; LVEI – left ventricular eccentricity index; AUC – Area Under Curve.

| 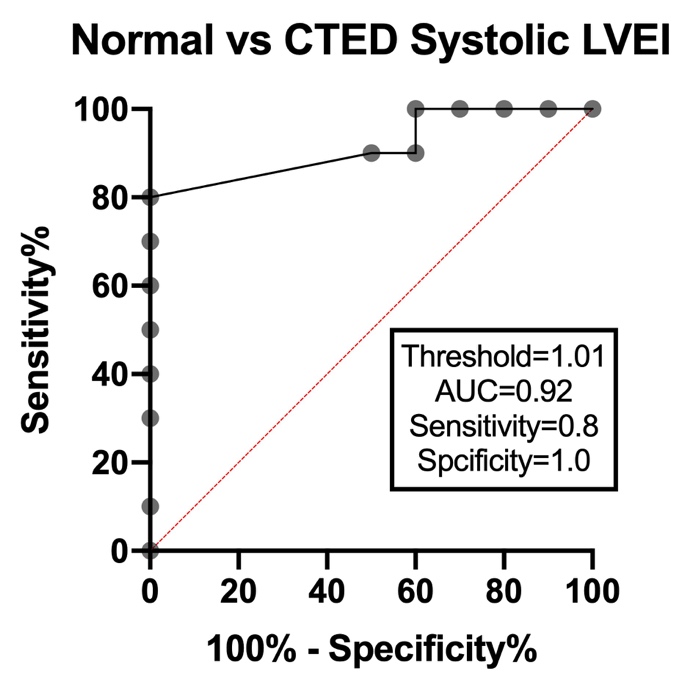 | 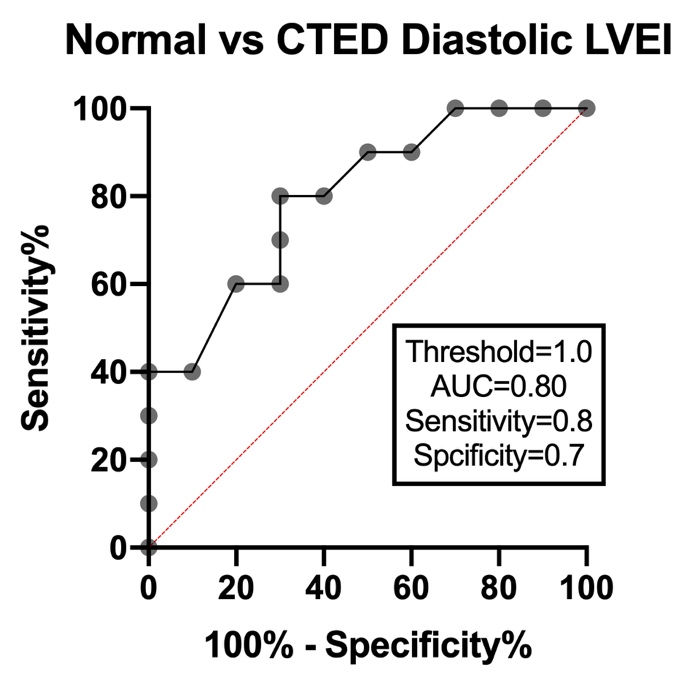 |
| --- | --- |
| 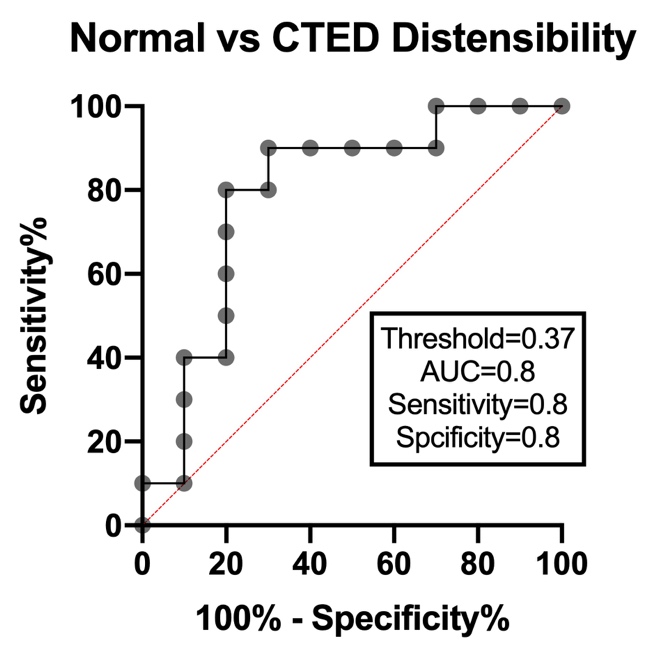 | 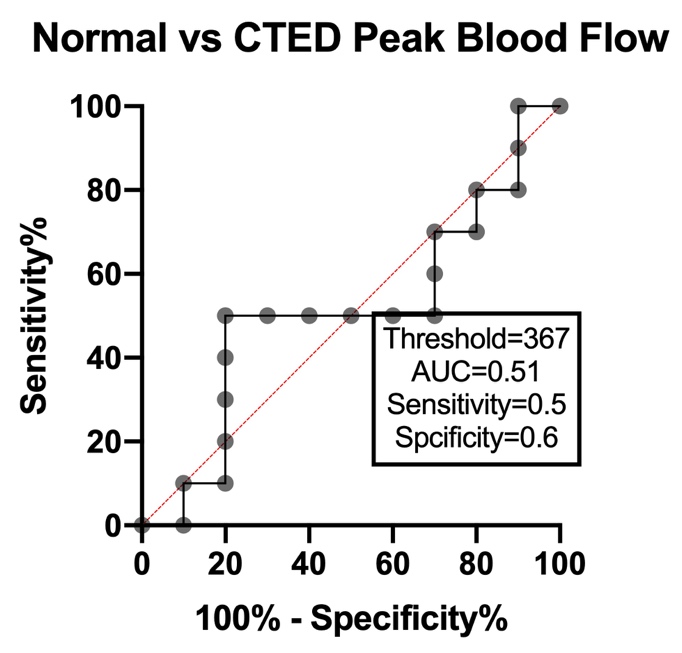 |
